# Building photonic links for microwave quantum processors

**DOI:** 10.1515/nanoph-2024-0599

**Published:** 2025-02-07

**Authors:** Han Zhao

**Affiliations:** Department of Physics, 6243 University of Central Florida, Orlando, FL, 32816, USA

**Keywords:** quantum network, hybrid quantum devices, quantum transduction, frequency conversion, optically heralded entanglement

## Abstract

Optical photons play unique role in transmitting information over long distances. Photonic links by the optical fiber networks compose the backbone of today’s global internet. Such fiber optics can also provide the most cost-effective quantum channels to distribute quantum information across distant stationary nodes in future large-scale quantum networks. This prospect motivates the recent emerging efforts in developing microwave-optical quantum transduction technology to interconnect microwave quantum processors. Various frequency conversion approaches are investigated to efficiently bridge the enormous electromagnetic frequency gap while preserving quantum coherence. Nonetheless, high-fidelity entanglement generation between remote quantum processing units has remained out of reach to date. Here, we summarize the state-of-the-art progresses in quantum transducer engineering and provide the perspectives on the key challenges and opportunities toward optically heralded quantum entanglement distributions.

## 1. Introduction

The prospect of “quantum internet” has inspired global interests in building distributed quantum networks where a plethora of remote stationary quantum processor nodes can work synergistically for complex quantum information processing [1], [2]. At the physical layer, the key enabling component of such quantum networks is the interconnects that bridge different quantum systems. To this end, optical photons, particularly in the telecommunication band, play unique roles as the media for transmitting quantum information over long distances owing to the low propagation loss via the global fiber infrastructure and the excellent isolation of thermal decoherence at room-temperature ambient [3]. To date, a few demonstrations have reported success in entangling remote quantum systems with intrinsic optical transitions, proving the viability of optical quantum networking [4], [5], [6], [7], [8].

On the other side of the electromagnetic spectrum, microwave photons are frequently employed as the carriers for quantum processors. Critically, superconducting qubits, known as the synthetic quantum two-level systems in superconducting microwave circuits, have become one of the leading technologies for quantum computing [8], [9], [10]. Quantum networking of superconducting qubits will allow for unprecedented scalability to overcome the hardware overhead for quantum error corrections [11], [12], [13], [14], [15]. However, these qubits typically work with transition frequencies in the 3–10 GHz range, which require low temperature environment at ∼10 mK to avoid thermal decoherence. While microwave photon-mediated quantum entanglements at meter-long separation have been demonstrated with extensive cryogenic engineering [16], [17], the practical solution for the quantum counterpart of large-scale cloud computing requires efficient quantum microwave-optical interfaces [18].

Transducing quantum information from microwave to optical frequencies and vice versa, therefore, has become an important active research area in quantum photonic engineering [19], [20], [21]. The five-order-of-magnitude frequency gap along with the absence of strong interactions at the single-photon level calls for surging efforts to address many open questions. This Perspective aims to provide a comprehensive introduction to the field of quantum transduction and its broader importance in building quantum processor networks. We begin with the most relevant metrics for gauging the performance of a quantum transducer within the context of remote superconducting qubit entanglement generations. After reviewing the state-of-the-art implementations, we highlight the key challenges and possible tackling solutions that can push the boundaries of the transduction performance. Finally, we provide an outlook on the future steps toward the experimental realizations of optically heralded entanglement and hybrid quantum repeaters – the two fundamental building blocks for quantum processor networks.

## 2 Metrics for microwave-optical quantum transduction

Fast and high-fidelity entanglement generation between remote superconducting qubits usually translates to the achievement of quantum transducers with *high conversion efficiency*, *low added noise*, and *large operation bandwidth* [22]. For distributed quantum computing applications, high connectivity between multiqubit quantum processors also requires *scalability* of the implementations [23], [24]. These factors make the design of quantum transducers a multiobjective optimization problem. We will see in the following sections that a deep understanding of the interplay among these metrics is crucial for engineering of a quantum transducer.

### 2.1 Conversion efficiency

The ratio of the converted output optical/microwave photons over the input microwave/optical photons in the transducer’s bandwidth defines the efficiency of a quantum transducer (*η* = *N*_out, o(e)_/*N*_in, e(o)_). A subunitary efficiency represents loss of the quantum information decaying through unwanted channels. Although high efficiency is generally favored, the hierarchy of the role is dependent on the exact entanglement protocol. For example, remote deterministic entanglement can be created by controlled swapping of a single quantum excitation between two qubits connected via a quantum channel [25], [26]. If the channel is to be implemented via microwave-optical transducers, near-unitary conversion efficiency is highly desirable because any loss of the quantum excitation translates to the infidelity of the entanglement process. A threshold of the efficiency can be found by considering the quantum capacity, that is, the maximal rate at which the quantum information can be transmitted coherently: since the quantum capacity is bounded by $Q=\max\left\{0,\log_{2}\frac{\eta }{1-\eta }\right\}$ , the prerequisite for nonzero quantum capacity is *η* > 1/2 [27]. In contrast, in heralded entanglement schemes where the generation of entanglement is created on the success of single-photon detections, a reduction of conversion efficiency does not compromise the fidelity of entanglement, as we will discuss in the last section. Instead, the conversion efficiency becomes a determining factor on how fast the entanglement can be successfully heralded [22], [28], [29], [30].

### 2.2 Added noise

Microwave-optical frequency conversion is typically accompanied by excessive noise from the simultaneous conversion of the thermal occupations. The origin of such noise can generally be traced to the finite bath temperature and the absorption of optical/microwave pumps required in various conversion approaches [31], [32], [33], [34], [35]. To model the transducer’s noise performance as an added noise source, the output optical/microwave noise photons (*n*_out_) are usually referenced to the input microwave/optical port, defining the input-referred added noise (*n*_add_ = *n*_out_/*η*). Intuitively, this quantity represents the equivalent noise photon number added to the input port that generates the same output noise quanta. The added noise is a major source of quantum decoherence, which undermines the fidelity in all kinds of entanglement schemes, therefore, warrants design strategies for mitigation. To date, the noise level of the state-of-the-art transduction techniques remains far from allowing quantum error-correction below error rate threshold (∼10^−3^ for surface code). Nonetheless, much recent effort in transducer engineering has been devoted to achieving subphoton input-referred added noise (*n*_add_ < 1), referred to as the “quantum-enabled” regime for that the transduced single-photon excitation dominates over the added thermal noise quanta [36], [37].

### 2.3 Bandwidth

The upper limit of the speed at which the transducer can be operated becomes an important consideration when coupling a superconducting qubit to the quantum transducer. Because of the finite lifetime of superconducting qubits, the frequency conversion should happen before the decay and decoherence of the quantum excitation. This sets the requirement for the bandwidth of the quantum transducer at the reciprocal of the decay and dephasing time constants of the qubits. A quantum transducer operated at a higher bandwidth can also speed up the single-photon flux output and thereby boost the success rate of remote entanglement.

### 2.4 Scalability

Many foreseeable applications of distributed quantum computing require high connectivity among multiqubit quantum processors. To this end, integrated quantum transducers with compatibility to planar superconducting qubit architectures will enable multiple optical quantum links due to the low device profile and heat load [38], [39], [40], [41].

## 3 Approaches for microwave-to-optical quantum frequency conversion

### 3.1 Direct cavity electro-optic transduction

**Figure 1:**
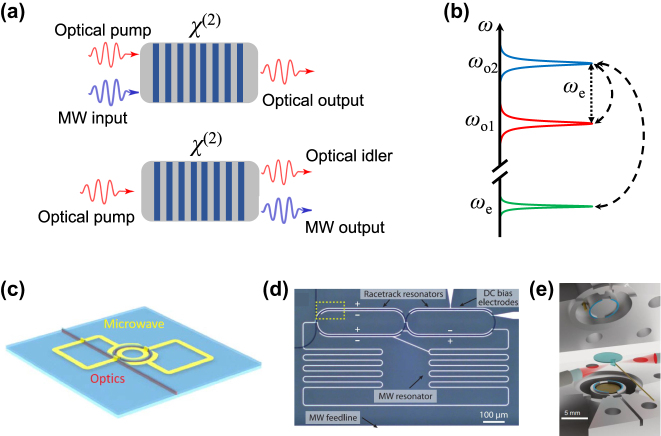
Electro-optic quantum transduction. (a) Schematic illustration of three-wave mixing (*χ* ^(2)^) processes for electro-optic up-conversion (upper panel) and down-conversion (lower panel). MW, microwave. (b) Scattering picture of the triple-resonance condition. For an efficient electro-optic frequency conversion, the microwave and the two optical frequencies coincide with resonances, respectively. The difference of the two optical frequencies should match the microwave frequency. (c) A cavity electro-optic frequency converter using single-crystal aluminum nitride [43]. The two optical resonances are the spectrally adjacent transverse-electric (TE) mode and transverse-magnetic (TM) mode of a single optical ring resonator. (d) A thin-film lithium niobate (LN) electro-optic frequency converter [46]. Two evanescently coupled optical ring resonators lead to two hybridized optical modes separated by the microwave frequency. (e) A bulky electro-optic transducer with a bulk LN optical resonator and a three-dimensional microwave cavity [47]. The optical resonances are provided by two cavity modes separated by a free spectral range.

The interconversion between microwave and optical photons can be realized by the electro-optic *χ* ^(2)^ nonlinearity [42]. To bridge the frequency gap, a pump at optical frequency is necessary, composing the picture of three-wave mixing (Figure 1(a)). To benefit from the cavity enhancement, the process is usually accomplished under the triple-resonance condition where all microwave/optical photons are on resonance and the frequency difference of the optical pump and signal matches exactly with the frequency of microwave resonance (Figure 1(b)). The optical resonances can be supported by either different modes in a single cavity (Figure 1(c)) [43], [44] or the hybridized modes in a dual-cavity photonic molecule (Figure 1(d)) [45], [46]. The conversion mechanism can be understood by the Pockel effect: the time-varying electric field modulates the optical refractive index, which leads to fast oscillation of the optical resonances. The photon conversion efficiency is given by [20]:

$$\eta =\eta_{\text{o}}\eta_{\text{e}}\frac{4C_{\text{eo}}}{{\left(1+C_{\text{eo}}\right)}^{2}},$$

where *η*_e(o)_ = *κ*_ext, e(o)_/*κ*_e(o)_ is the extraction efficiency of the microwave (optical) cavity, and *C*_eo_ = 4*n*_c_ *g*_eo_^2^/*κ*_o_ *κ*_e_ is the electro-optic cooperativity, with *n*_c_ being the intracavity photon number of the pump and *κ*_e(o)_ being the decay rate of the microwave (optical) cavity. The single-photon electro-optic coupling coefficient (*g*_eo_) is a function of the *χ* ^(2)^ nonlinearity as well as the spatial mode overlap between microwave and optical fields in the Pockel material, which can be expressed as

$$\begin{array}{ll} g_{\text{eo}} & =\sqrt{\frac{\hbar }{2\varepsilon_{0}}\prod_{m=a,b,c}\frac{\omega_{m}}{\int dV\sum_{ij}\varepsilon_{ij}{ē}_{i,m}{ē}_{j,m}}}\int_{\text{Pockel}}\sum_{ijk}\chi_{ijk}^{\left(2\right)}{ē}_{i,a}{ē}_{j,b}^{*}{ē}_{k,c}dV. \end{array}$$

Here, *i*, *j*, *k* are the indices for the spatial coordinates, *a* and *b* represent the two optical modes, *c* represents the microwave mode, $\chi_{\mathrm{ijk}}^{\left(2\right)}$ is the second-order susceptibility tensor, and ${\bar{e}}_{\mu ,\nu }$ is the normalized zero-point complex field amplitude. The resulting input-referred added noise for microwave-to-optical upconversion and optical-to-microwave donwconversion are, respectively, related to the microwave thermal bath occupation *n*_th_ by:

$$n_{\text{add},\text{e}-\text{o}}=\frac{\eta_{\text{e}}}{1-\eta_{\text{e}}}n_{\text{th}},\;n_{\text{add},\text{o}-\text{e}}=\frac{1-\eta_{\text{e}}}{C_{\text{eo}}\eta_{\text{o}}}n_{\text{th}}.$$

Here, the microwave thermal bath occupation is a result of the thermalization to the ambient cryogenic environment and the pump-induced heating dynamics.

The performance of an electro-optical quantum transducer is critically dependent on the cooperativity *C*_eo_, which has been limited by the lack of strong electro-optical nonlinearity, the large discrepancy of microwave and optical mode volumes preventing significant mode overlap and the various loss mechanisms in the optical cavities/superconducting microwave resonators. Considerable research effort has been focused on pushing the electro-optic cooperativity to unity (i.e., *C*_eo_ → 1). To improve the single-photon electro-optic coupling, various material platforms with large intrinsic *χ* ^(2)^ nonlinear coefficients have been explored, with thin-film lithium niobate (LN) standing out as a preferred candidate [39], [44], [45], [46]. Careful microwave/optical mode engineering has also been attempted to obtain the maximal modal overlap while avoiding significant electrode-induced optical loss and light-induced quasi-particle generation in the superconductors. To date, *g*_eo_/2π ∼ 1 kHz has been achieved with on-chip optical ring resonators [39]. The recent progress in LN nanofabrication techniques allows routine realizations of high-quality optical resonators with *Q* ∼ 10^7^ [48], [49] corresponding to *κ*_o_/2π = *f*_o_/*Q* ∼ 20 MHz (*f*_o_ is the optical frequency). Despite these improvements, reaching the low-noise (*n*_add_ < 1) quantum regime has been challenging for integrated electro-optic approaches. A recent demonstration using a millimeter-scale bulk LN resonator has achieved low input-referred added noise of 0.16–0.4 with efficiency of 8.7 %–15 %, benefiting from the high intracavity pump photon number and the power handling capacity [36], [50] (Figure 1(e)). However, the low-noise operation relies on lowing the repetition rate of the pump pulses (<10 Hz), limiting the effective bandwidth of the transducer.

### 3.2 Mediated quantum transduction

Microwave-optical frequency conversion can also be achieved via a third entity in between. Coupling photons to other degrees of freedom can leverage the unique advantages of the mediators, including larger cooperativity at the single-photon level, higher tunability and spectral uniformity.

**Figure 2:**
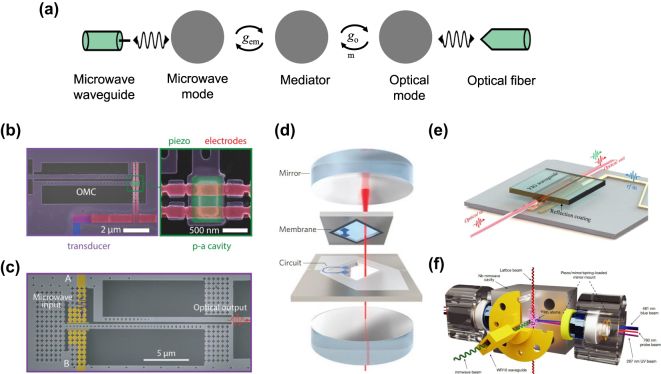
Microwave-optic quantum frequency conversion via a mediator. (a) Schematic of mediated frequency conversion between microwave and optical photons. The mediator couples to both microwave and optical photons at the single-photon coupling rate of *g*_em_ and *g*_om_, respectively. (b) A piezo-optomechanical transducer [59]. The electromechanical coupling is realized by an interdigital transducer on a heterogeneous stack of piezoelectric aluminum nitride and single-crystal silicon. The optomechanical coupling is realized in the optomechanical crystal cavity (OMC). (c) An integrated electro-optomechanical transducer in single-crystal silicon [65]. Instead of using piezoelectric materials, the electromechanical coupling is realized by electrostatic actuation at a parallel-plate capacitor (pseudo-colored in yellow). (d) An electro-optomechanical transducer implemented with a high-Q silicon nitride mechanical membrane resonator in the middle of an optical Fabry–Perot cavity [66]. (e) An integrated optomagnonic frequency converter with yttrium iron garnet (YIG) optical waveguide [38]. (f) A millimeter-wave-to-optical frequency converter assisted by Rydberg atoms [37].

One of the most promising mediators is mechanical oscillators [51], [52], [53], [54], [55], [56], [57], [58], [59], [60], [61], [62], [63], [64]. In this scenario, the frequency conversion is a two-step process where the microwave photons are converted to mechanical oscillation via electromechanical coupling and subsequently to optical photons via optomechanical coupling and vice versa (Figure 2(a)). Assuming the microwave cavity is on-resonance with the mechanical resonator and the optical pump is at the red/blue sideband of a sideband-resolved optical cavity, the transduction efficiency is given by

$$\eta =\eta_{\text{o}}\eta_{\text{e}}\frac{4C_{\text{em}}C_{\text{om}}}{{\left(1+C_{\text{em}}+C_{\text{om}}\right)}^{2}},$$

where *C*_em_ = 4*g*_em_^2^/*κ*_e_ *κ*_m_ is defined as the electromechanical cooperativity with the electromechanical coupling *g*_em_ and the mechanical decay rate *κ*_m_, *C*_om_ = 4*n*_c_ *g*_om_^2^/*κ*_o_ *κ*_m_ is the optomechanical cooperativity with the single-photon optomechanical coupling *g*_om_. In addition to the microwave thermal bath (*n*_e_), the added noise for mechanics-mediated transduction can also be sourced from the mechanical thermal bath (*n*_m_) as:

$$\begin{array}{ll} n_{\text{add},\text{e}-\text{o}} & =\frac{\eta_{\text{e}}}{1-\eta_{\text{e}}}n_{\text{e}}+\frac{1}{C_{\text{em}}\eta_{\text{e}}}n_{\text{m}},\\ n_{\text{add},\text{o}-\text{e}} & =\frac{1-\eta_{\text{e}}}{\eta_{\text{o}}}n_{\text{e}}+\frac{1}{C_{\text{om}}\eta_{\text{o}}}n_{\text{m}}. \end{array}$$

Strong electromechanical coupling can be conveniently implemented with piezoelectric materials such as aluminum nitride [52], [58], [59], lithium niobate [60], [61], [62], gallium arsenide [53], and gallium phosphide [63], [64], allowing near-unity conversion between microwave photons and gigahertz mechanical oscillation. On the other side, the optomechanical coupling can be realized by the radiation pressure exerted on the mechanical resonator by the intracavity optical photons [67]. Owing to the similar wavelength scale of the gigahertz mechanical displacement field and the optical field, *g*_om_/2π as high as ∼1 MHz has been achieved in suspended photonic crystal cavities using single-crystal silicon [68], which is three orders of magnitude compared to the electro-optic counterpart. The piezo-optomechanical approach has been experimentally explored extensively on piezo-silicon heterogeneously integrated platforms, realizing *n*_add_ ≈ 0.14 at *η* ≈ 10^−4^ [59] and *n*_add_ ≈ 1.6 at *η* ≈ 4 % [62] in two instances (Figure 2(b)). Due to the large piezoelectric coupling, bandwidth >1 MHz can be routinely attained in piezo-optomechanical transducers, although the actual converted photon flux is usually limited by the repetition of the pulsed operation.

Despite the demonstrations of low-noise operations, the mechanical resonators in piezo-optomechanical transducers suffer high mechanical loss, which greatly compromises the prospect of the approach. An alternative electrostatic actuation mechanism for gigahertz mechanical oscillation is, therefore, pursued on piezo-free single-crystal silicon platform, enabling an electro-optomechanical transducer with *n*_add_ < 1 at *η* ≈ 3 % [65], [69], [70] (Figure 2(c)). Owing to the reduced heating and the low intrinsic mechanical loss in single crystal (*Q*_m_ ∼ 10^7^) [71], this transducer operates with continuous-wave optical pump, utilizing the full bandwidth of 90 kHz. Another genre of piezo-free electro-optomechanical transducers features the electromechanical actuation of megahertz-frequency mechanical modes in silicon nitride membranes embedded in bulk Fabry–Perot optical cavities [33], [66], [72] (Figure 2(d)). The bulky structure allows large optical pump photon numbers that can be leveraged to engineer balanced electromechanical and optomechanical cooperativities for high transduction efficiency. Though essentially free from optical pump induced heating, the actuation of the MHz mechanical mode requires microwave pump leading to a hot microwave bath. A demonstration along this direction has shown continuous transduction with *n*_add_ ≈ 3 at *η* ≈ 47 %. However, the low optomechanical and electromechanical coupling rates have limited the transduction bandwidth to ∼200 Hz [33].

Similar to phonons in mechanical oscillators, magnons, i.e., the collective excitations of spins in magnetic materials, represent another candidate for mediating quantum transduction [38], [73], [74], [75], [76]. This approach can benefit from the vast tunability of the magnon resonances and the ultra-strong coupling with microwave photons [77], [78]. However, due to the mismatch of the magnon and optical mode volumes and the challenging nanofabrication of the magnetic materials, the single-photon magneto-optic coupling, in analogy to the single-photon optomechanical coupling *g*_om_, has been limited to ∼20 Hz [38] (Figure 2(e)). Efficient magnon-assisted transduction thus requires large pump power, which can result in excessive heating and added noise. The low-noise operation and possible noise mitigations of such transduction approach still warrant future investigations. Atom-assisted transduction exploits the intrinsic microwave and optical transitions to enable enhanced effective *χ* ^(2)^ nonlinearity [79]. Along this direction, many candidates including color centers in diamond [80], trapped ions [81], rare-earth-ion (REI) doped crystals [33], [82], [83], and Rydberg atoms [37], [84] have been developed. This approach can benefit from improved added noise performance due to reduced pump-induced heating. High internal transduction efficiency of *η* = 58 % with *n*_add_ = 0.6 and a bandwidth of 360 kHz has been demonstrated for the conversion between millimeter-wave photons and visible-range optical photons using ensemble of cold ^85^Rb atoms [37] (Figure 2(f)). Another attractive feature of atom-mediated transduction is the spectral uniformity of the transduced optical emission across different sample devices. This is particularly beneficial in ensuring nondistinguishability of the transduced single photons. A recent work on REI-based transducers has demonstrated coherent interference of the emitted optical photons from two simultaneously operated transducers [83].

## 4 Challenges toward the ideal quantum transducer

Despite the extensive efforts on all fronts of transduction approaches, an ideal quantum transducer satisfying all desirable metrics for remote superconducting qubit entanglement remains elusive. The state-of-the-art performance in terms of conversion efficiency and added noise has been far from the threshold of positive quantum capacity for deterministic entanglement generation [27], [85]. The heralding entanglement protocol, while with relaxed requirements on the transducer performance, calls for orders-of-magnitude enhancement in low-noise single-photon flux rate for practically useful remote quantum connectivity. To allow future improvements, it is important to first identify the challenges in the designs of quantum transducers.

An outstanding challenge faced by most quantum transducers is the absorption of the pump power necessary for parametric enhancement of the transduction efficiency. For the direct electro-optic transduction on integrated platforms, the requirement for large microwave and optical mode overlap necessitates proximity of the optical cavity and the capacitor electrodes of the superconducting microwave circuits. However, superconductors are well-known as being exceptionally susceptible to optical illumination, and slight absorption of optical photons can generate severe quasi-particles and elevated thermal population in the microwave resonator [34], [86]. This absorption not only induces added noise as the thermal microwave photons convert to optical photons but also causes microwave frequency shift and cavity linewidth broadening, which deteriorate the electro-optic cooperativity [87]. Photo-refractive effect is another challenge widely seen in thin-film LN transducers, where the optical photons traveling in LN waveguides create unwanted charge carriers that shift the optical field out of the resonance condition [45]. Mechanics-mediated transducers allow spatial separation of the microwave and optical fields by engineering distributed acoustic cavity modes, which can be used to avoid the direct optical illumination on superconductors. Nonetheless, substantial optical-absorption heating of the mediating mechanical resonator has been observed in piezo-optomechanical transducers, leading to both high mechanical thermal occupation and mechanical linewidth broadening [41], [59], [62]. This has limited low-noise operations to be only possible with low efficiency at ∼10^−4^ at weak optical pump power. The membrane-in-the-middle (MIM) electro-optomechanical approach, on the other hand, is essentially free from optical pump induced heating. However, the strong microwave pump required for the actuation of the MHz mechanical modes instead becomes the major source of heating in such systems [34].

Radiative cooling is one of the strategies to mitigate the heating problem. By engineering a decay channel to cold environmental baths, the excessive thermal occupation can dissipate via other nontransduction ports and thus be suppressed. For cavity electro-optic transducers thermalized to ∼10 mK temperature, radiative cooling can be realized by significantly overcoupling the microwave resonator to the waveguide (*η*_e_ = *κ*_ext_/*κ*_e_ ∼ 1) [88]. The mechanical thermal occupation in mechanics-mediated transducers can be radiatively cooled using either the electromechanical or optomechanical decay channels. For transducers with GHz mechanical modes, the microwave bath typically remains cold even with continuous optical pump, enabling radiative cooling via the electromechanical decay channel [89]. This motivates a recent work realizing electromechanical quantum ground-state cooling by exploiting an asymmetry in electromechanical and optomechanical decay rates combined with the narrow mechanical linewidth [65]. In contrast, optomechanical quantum ground-state cooling has been demonstrated for an MIM electro-optomechanical transducer [34].

Passive mechanical cooling through better thermal contact is proposed as another measure to suppress optical-absorption heating in transducers involving optomechanical crystal cavities. Instead of the suspended one-dimensional nanobeam clamped only at the two ends, two-dimensional optomechanical cavities allow heat dissipation via the 2D connections to the surrounding membrane, therefore, achieve lower thermal occupation at similar intracavity photon numbers [90], [91], [92]. Similarly, the concept of unreleased optomechanical cavity has also been proposed to accelerate the heat dissipation [93].

A more common mitigation to combat the heating problem is by pulsing the pump power. The heating dynamics usually contains a slow buildup process with finite characteristic rising time when the pump incidents on the otherwise cold transducer [34], [94]. If the pump duration is shorter than the “turn-on” time constant of the heating dynamics and the pause between adjacent pulses allows sufficient cooling to the cold environment, the thermal occupation can be suppressed below the equilibrium. Thereby, the transducer can benefit from a reduction of the added noise while maintaining the conversion efficiency. However, this improvement comes with an expense of significantly slower operation speed and lower output photon flux because of the low duty cycles necessary for the cooling.

Aside from the challenges intrinsic to the transducers, other technical barriers are also noteworthy for remote entanglement generation. For example, the optical on-chip coupling efficiency (from the optical output waveguide to the fiber) needs to be included in the overall transduction efficiency when considering the remote entanglement experiments. However, the fiber-chip alignment in a dilution refrigerator has been difficult due to thermal contraction and inaccessibility to optical imaging. On-chip grating couplers with vertical fiber array reach ∼10 % coupling efficiency at maximum [95]. End-fire coupling though a lensed fiber can reach fiber-waveguide coupling efficiency at 30 %–70 % but requires intensive efforts of alignment after cooling down the sample to the fridge base temperature [58], [59], [62], [65]. Several recent developments of chip-glued tapered fiber couplers show robustness despite the thermal contraction while reach 50 %–80 % coupling efficiency, lifting the necessity of the challenging fine alignment inside a dilution refrigerator [96], [97].

Additionally, single-photon detection of the transduced optical photons requires the filtering of the optical pump. Except the scenario of magnon-mediated transducers where the transduced photons are accompanied by a polarization rotation due to Faraday effect, high-fineness, high-extinction spectral bandpass filters are necessary for most microwave-optical quantum transducers. Suppressing the filter insertion loss is, therefore, important to preserve the precious output photon flux from the transducers.

The spectral uniformity of the transduced optical photons is a necessary condition for most remote entanglement protocols requiring indistinguishable photons. Due to limited nanofabrication precision, however, the intrinsic resonance frequencies of two “identical” optical cavities in design typically have frequency variations at ∼100 GHz level, which implies frequency mismatch of the transduced optical photons. On-demand tuning of optical resonance is, therefore, an important technology to overcome this limitation. A direct *in situ* tuning of silicon nanophotonic cavity resonance has been demonstrated at low temperature via electrical field-induced nano-oxidization [98]. Additionally, recent advance of LN electro-optic modulators can readily cover the 100-GHz bandwidth requirement to compensate for the frequency variation, although the insertion loss of such modules would be lumped into the overall transduction efficiency [99], [100], [101]. Optical photon emission from atom-mediated transducers can be highly uniform because of the identical intrinsic transition energy across different samples. However, since the emission are typically in the visible range, the subsequent optical frequency down-conversion required for the telecom-band optical fiber network poses similar challenge of compromised transduction efficiency. This challenge further motivates the search for atomic systems readily operating at telecom-band optical transitions such as Er^3+^-doped crystals and color centers in silicon [102], [103], [104], [105], [106].

Beyond improving the performance of the transducers, another necessary engineering task is the seamless integration with superconducting qubits. To this end, initial attempt has been made to integrate a transmon qubit with a piezo-optomechanical transducer on the AlN-silicon heterostructure platform [58]. However, the optical pump illumination, predominantly the intracavity photons, created excessive quasi-particles that pollute the superconducting qubit. To overcome the limitation, modular packaging design that isolates the qubit module from the optical illumination is important in preserving the quantum coherence of the superconducting qubits.

## 5 Entangling remote superconducting qubits using quantum transducers

The main functionality of quantum transducers is to serve as a microwave-optical interface to entangle remote stationary superconducting qubits via flying optical photons. Due to the low conversion efficiency of the available transducers in the low-noise limit, short-term implementations for remote entanglement generation will rely on heralding schemes from fidelity considerations. As the first step toward the goal, several recent low-noise transducers have been exploited to probabilistically entangle microwave and optical fields [107], [108].

Among various remote entanglement schemes, a particularly suitable approach with current low-noise, low-efficiency transducers is the well-known Duan–Lukin–Cirac–Zoller protocol [28], [29]. In this picture, two identical copies of superconducting qubit-transducer nodes at a distance are connected to a beam splitter. The output ends of the beam splitter are both attached to single-photon detectors. The event of transduction at either node creates a single flying optical photon and a transition between the ground and the excited states of the stationary qubit. Here, because of the low transduction efficiency, we assume the probability of simultaneous transduction at both nodes is negligible and only one single photon can be transduced at one time tag. Thus, one click registered at either of the single-photon detectors heralds a transduction event at one of the two qubit-transducer systems. If the single-photon emission from the two transducers is indistinguishable, the path information of the detected photon is erased, that is, the transduction event can happen at either side with equal probability. Thereby, for the two remote qubits initiated at the same state, the single-photon detection projects the two-qubit state to the Bell-like state $\left(\left|e,g\right\rangle +e^{-i\varphi }\left|g,e\right\rangle \right)/\sqrt{2}$ , where $\left|e\left(g\right)\right\rangle$ represents either of the superconducting qubits is at the excited (ground) state.

**Figure 3:**
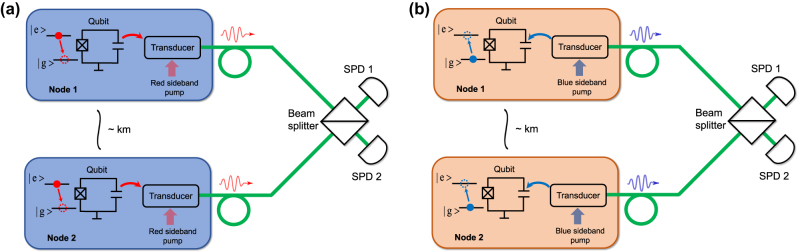
Optically heralded entanglement between two superconducting qubits at kilometer-long distances. (a) Heralding scheme based on microwave-to-optical state transfer. (b) Heralding scheme based on microwave-optical photon pair generation.

The exact scheme of the above protocol can be implemented in two configurations, depending on the operation of the transducers. One of them is to operate the transducer with a red-sideband pump (optical pump frequency lower than the optical resonance), which allows the transfer of the qubit excitation to an optical photon, thus termed the *microwave-to-optical state transfer mode*. The transducers can also be operated with a blue-sideband pump (optical pump frequency higher than the optical resonance), where the transduction generates a pair of microwave and optical photons via spontaneous down-conversion, which is the *photon-pair generation mode*. With the transducers operating at the microwave-to-optical state transfer mode, both superconducting qubits are initiated at the excited state (Figure 3(a)). A transduction event up-converts the microwave qubit excitation to a single optical photon, leaving the qubit at the ground state. Since the generation of a single transduced photon is always associated with a $\left|e\right\rangle \to \left|g\right\rangle$ transition at one of the qubits, a single-photon detection heralds the entanglement of the two superconducting qubits ( $\left|e,e\right\rangle \to \left(\left|e,g\right\rangle +e^{-i\varphi }\left|g,e\right\rangle \right)/\sqrt{2}$ ) as the path information is erased by the beam splitter. Conversely, in the photon-pair generation mode, both superconducting qubits are initiated at the ground state (Figure 3(b)). In this case, the detection of a down-converted optical photon heralds the generation of a microwave excitation, which induces a $\left|g\right\rangle \to \left|e\right\rangle$ transition at one of the qubits. Thereby, the detection of a single transduced photon conditions the two-qubit state change $\left|g,g\right\rangle \to \left(\left|e,g\right\rangle +e^{-i\varphi }\left|g,e\right\rangle \right)/\sqrt{2}$ .

As discussed earlier, the success rate and fidelity of such entanglement generation protocols are critically dependent on the performance of the transducers [22]. The rate at which one single-photon detector sees a transduced photon is proportional to output photon flux determined by the product of the conversion efficiency, bandwidth, and repetition rate (for pulsed mode). It is worth noting that the photon loss due to nonunitary conversion efficiency does not lead to the infidelity of entanglement generation because the photon detection heralding is unidirectional. However, the added noise at the transducer output is usually a dominant contributor to the infidelity of the entanglement. The exact roles of the added noise in the aforementioned two entangling schemes are different: in the microwave-to-optical state conversion mode, the optical added noise arriving at the single-photon detectors registers “false” clicks at which the heralding mechanism does not apply; in the photon-pair generation mode, the microwave added noise, which is not correlated at the two qubit-transducer nodes, can lead to decoherence of the qubits. Additionally, we note that, in both scenarios, if the photon detector cannot distinguish the photon number, infidelity can also be resulted from the false clicks when the transduction happens at the two qubit-transducer nodes simultaneously and one of the photon detectors picks up two-photon states. While the probability of simultaneous transduction is negligibly low for two transducers with low conversion efficiency (as we have assumed in the previous discussion), such false clicks become considerable as the transduced photon flux improves, leading to a trade-off between fidelity and speed in the longer-term developments.

Looking a further step ahead, although remote entanglement mediated by telecom-band optical fibers can be reliably sustained for as long as ∼10 km, quantum links over longer distances would require the presence of quantum repeaters to relay the long-haul transmission of quantum information. Such a quantum repeater should have an efficient optical-spin interface to allow optically heralded spin-qubit entanglement and long quantum memory to store the entangled states. To this end, atomic systems featuring long spin coherence time, such as silicon-vacancies in diamond [106], rare-earth-doped crystals [109], cold atoms [110], and color centers in silicon [104], [105], are, therefore, ideal candidates. Remarkably, a recent demonstration has achieved a spin-photon interface in the strong coupling regime by engineering high quality-factor nanophotonic cavities in diamond [111]. Combining an additional optical frequency conversion process [7], a quantum repeater protocol for long-range quantum networks can be envisioned.

## 6 Conclusions

Microwave-optical quantum transduction is the indispensable technology for distributing entanglement among remote microwave quantum processors. The pushing of quantum transducer performance on all fronts of metrics, i.e., high efficiency, low added noise, large bandwidth, and high scalability, have direct impact on the prospect of scaling superconducting qubits through quantum networks. Despite the increasing endeavor on all kinds of quantum transducer platforms, several expectations, such as higher cooperativity at single-photon pump, better heat dissipation, as well as high-efficiency packaging and light-robust integration with superconducting qubits, pose rewarding opportunities to quantum transducer engineering. Moving forward, near-term improvements of many state-of-the-art quantum transducer modalities can be readily sufficient to enable several optically heralded schemes for entangling remote superconducting qubits, which will mark an important milestone toward quantum processor networks and distributed quantum computing. Another possible application of quantum transducers is using superconducting qubits to generate and control flying photonic qubits and nonclassical optical states that are otherwise difficult to obtain for optical quantum computing [112]. Lastly, in addition to interfacing remote quantum systems, we note several recent works explore the benefits of using efficient microwave-optical frequency converters for microwave control and readout of superconducting qubits [113], [114], [115], [116]. The exceptionally low heat load and low cost of fiber optics may provide an attractive approach to scale up the microwave control hardware for large-scale superconducting quantum processors.
